# Development of a preclinical humanized mouse model to evaluate acute toxicity of an influenza vaccine

**DOI:** 10.18632/oncotarget.25399

**Published:** 2018-05-25

**Authors:** Eita Sasaki, Haruka Momose, Yuki Hiradate, Keiko Furuhata, Takuo Mizukami, Isao Hamaguchi

**Affiliations:** ^1^ Department of Safety Research on Blood and Biological Products, National Institute of Infectious Diseases, Musashi-Murayama, Tokyo 208-0011, Japan

**Keywords:** influenza vaccine, humanized mouse, biomarker, safety evaluation, human peripheral blood mononuclear cell, Immunology

## Abstract

Safety evaluation of a human vaccine is critical for vaccine development and for preventing an unexpected adverse reaction in humans. Nonetheless, to date, very few systems have been described for preclinical studies of human adverse reactions *in vivo*. Previously, we have identified biomarker genes expressed in the lungs for evaluation of influenza vaccine safety, and their usefulness in rodent models and for adjuvant-containing vaccines has already been reported. Here, our purpose was to develop a novel humanized mouse model retaining human innate-immunity–related cells to assess the safety of influenza vaccines using the previously identified biomarker genes. In the present study, we tested whether the two humanized models, a short-term and long-term reconstitution model of NOD/Shi-scid *IL2rγ^null^* mice, are suitable for biomarker gene–based safety evaluation. In the short-term model, human CD14^+^ cells, plasmacytoid dendritic cells, CD4^+^ and CD8^+^ T cells, and B cells were retained in the lungs. Among these cells, human CD14^+^ cells and plasmacytoid dendritic cells were not detected in the lungs of the long-term model. After the vaccination, the expression levels of human biomarker genes were elevated only in the short-term model when the toxicity reference vaccine was inoculated. This phenomenon was not observed in the long-term model. The levels of human cytokines and chemokines in the lungs increased in response to the toxicity reference vaccine in the short-term mouse model. According to these results, the short-term model provides a better platform for evaluating vaccine safety in terms of human peripheral blood mononuclear cell–mediated initial reactions *in vivo*.

## INTRODUCTION

Vaccination is an effective method for prevention of infectious diseases and has been widely used by many people around the world. Vaccines are inoculated into healthy people, including children and elderly subjects. Therefore, a safety evaluation system is needed to predict or evaluate adverse reactions caused by vaccines in humans.

Adverse reactions caused by vaccination, especially pain at the site of inoculation, swelling, and fever, are thought to be due to the initial biological response caused by an endotoxin, vaccine antigen, or adjuvants [1]. These reactions often occur within 1 day of inoculation. To predict these reactions, tests for detection of acute toxic reactions such as a pyrogen test, abnormal toxicity test (ATT, also known as the general toxicity test), and the leukopenic toxicity test, are implemented according to the Minimum Requirements for Biological Products Guidelines of Japan (JMR) [2], and similar tests have been implemented in other countries according to their guidelines. To evaluate these toxic reactions to vaccination, we identified the biomarkers for evaluation of toxicity in rodent models [3]. In particular, we found that approximately 18 genes expressed in rat lungs are useful for the safety assessment of influenza vaccines [4–6]. Among these genes, an increase in expression levels is observed when the animals are vaccinated with a whole-particle inactivated influenza vaccine [3], for which pain at the site of inoculation, fever, and swelling are observed at a relatively high frequency. Nonetheless, when a hemagglutinin split vaccine, which is associated with relatively few reports of adverse reactions, is inoculated, the above increase in expression is not observed [3]. As mentioned above, these genes have been expected to serve as safety assessment markers in the future, but the evaluation of their applicability to humans remains inadequate.

To date, very few systems have been described for preclinical research that reproduce human reactions *in vivo*. Humanized mouse models have been expected to serve as a useful preclinical tool for assessment of immune responses *in vivo* [7], but characterization of immune responses and toxicity for initial vaccination responses has not been reported. Engraftment of human peripheral blood lymphocytes (PBLs) in NOD/*SCID/IL2rγ*KO (NOG and NSG) mice has been reported involving either human CD34^+^ stem cells or peripheral blood mononuclear cells (PBMCs) [8–9]. In these methods, antigen-presenting cells, as typified by macrophages, monocytes, and dendritic cells (DCs), have not been engrafted [10–11]. Nevertheless, some researchers have succeeded in establishing human plasmacytoid DC (pDC)-retaining humanized mouse models [12–13]. These models are created by transplantation of CD34^+^ cells, and therefore, it takes a long time to prepare such a model. Our aim was to create a tool for evaluation of vaccine safety (in terms of the above-mentioned initial biological reactions to vaccination) as a simple method. Martino *et al.* reported that intravenously injected human PBMCs are transported into lungs within a few hours [14], and Wada *et al.* reported that immunodeficient NOJ (NOD/Scid/*Jak3^null^*) mice that were injected with human PBMCs at 24 h prior to the vaccination showed elevation of vaccination-dependent production of antigen-specific IgG, suggesting that APCs are recruited within 24–48 h after PBMC engraftment [15]. As stated above, there is a possibility that pDCs and monocytes may be retained within 24 h after engraftment, and this method would help to create a humanized model for assessment of influenza vaccine safety.

In this study, we attempted to evaluate influenza vaccine safety in humanized mouse models to demonstrate applicability of the above-mentioned marker genes to humans. In the 2000s, a series of immunodeficient mice was developed by introducing the *IL-2Rγ^null^* mutation into conventional SCID and *Rag1/2^null^* mice. These strains showed extremely high engraftment rates and differentiation of human cells, resulting in remarkable advances in the development of human disease models. Data accumulated to date suggest that NOG/NSG mice are the best recipients of a humanized tissue, and the success of human-cell engraftment can be ranked in the following order: NSG = NOG > NOD.Cg-*Rag ^tm1Mom^ Il2rg^tm1Wjl /SzJ^* (NRG) > BALB/c-*Rag2^null^ Il2rg^null^* (BRG) > NOD.CB17*-Prkdc^scid^*/J (NOD/SCID) > C57BL/6-*Rag2^null^ Il2rγ^null^* (B6RG) [16, 17]. Therefore, we chose NOG mice as recipients to develop the humanized mouse model. Judging by the results, we discuss the usefulness of the resulting humanized mouse models for reproducing the human early immune response *in vivo.* We show that the marker genes of the influenza vaccine safety can predict adverse reactions in humans.

## RESULTS

### Development of humanized mouse models

**Figure 1:**
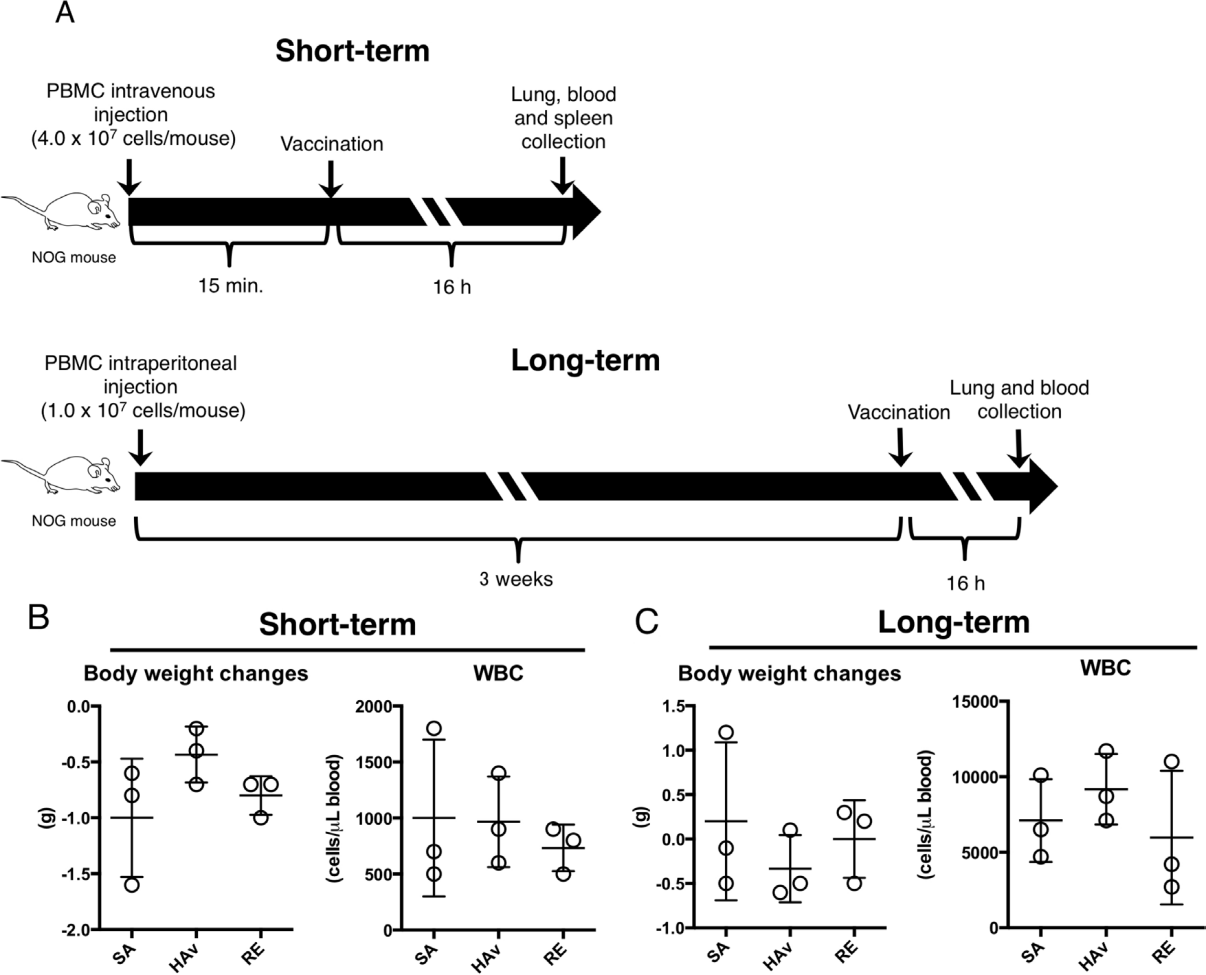
Creation of two humanized mouse models and effects of vaccination on body weight changes and WBCs (**A**) As shown in the experimental scheme for humanized mouse models indicated as “short-term” and “long term” models, the two humanized mouse models were applied to the vaccination study, and 16 h after the vaccinations, body weight and numbers of WBCs were assessed. The data are shown for the short-term (**B**) and long-term model (**C**). Each circle represents a result from an individual animal, and horizontal bars denote mean ± SD.

Before using PBMCs, we analyzed the composition of lymphocytes (T cells and B cells), monocytes, and pDCs by fluorescence-activated cell sorting (FACS). The frequencies of cell types were 52% for CD4^+^ T cells, 7.8% for CD8^+^ T cells, 5.0% for CD19^+^ B cells, 17.7% for monocytes (CD14^+^ cells), and 0.11% for pDCs among all PBMCs (Figure 1A and 1B) in agreement with the reported normal PBMC phenotype [18].

To develop humanized mouse models, human PBMCs were intravenously injected into NOG mice at 2.0 × 10^7^ cells/mouse as described in another study [14]; this model was named the “short-term model (ST-model).” In another model, human PBMCs were intraperitoneally injected into NOG mice at 10^7^ cells/mouse, and the model was named the “long-term model (LT-model).” The schemes of the experiments are depicted in Figure 1A. Before the PBMC engraftment, subsets of lymphocytes, monocytes, and pDCs were analyzed to confirm quality of the human PBMCs (Supplementary Figure 1). The vaccinations were performed at 15 min after the PBMC transplant, and the end point of testing was set to 16 h after vaccination in the ST-model (Figure 1A). The end point was set according to studies showing a decrease in body weight and the white blood cell (WBC) number after vaccination [5, 19–20]. The LT-model, which is known to show good T-cell engraftment [11], was produced via 3 weeks of breeding after intraperitoneal PBMC engraftment (Figure 1A). The end point of testing was set to 16 h after vaccination, as in the ST-model (Figure 1A). Both body weight and numbers of WBCs were not significantly altered by the vaccination in both models (Figure 1B and 1C).

### Blood biochemical analyses and histopathological examination

**Figure 2:**
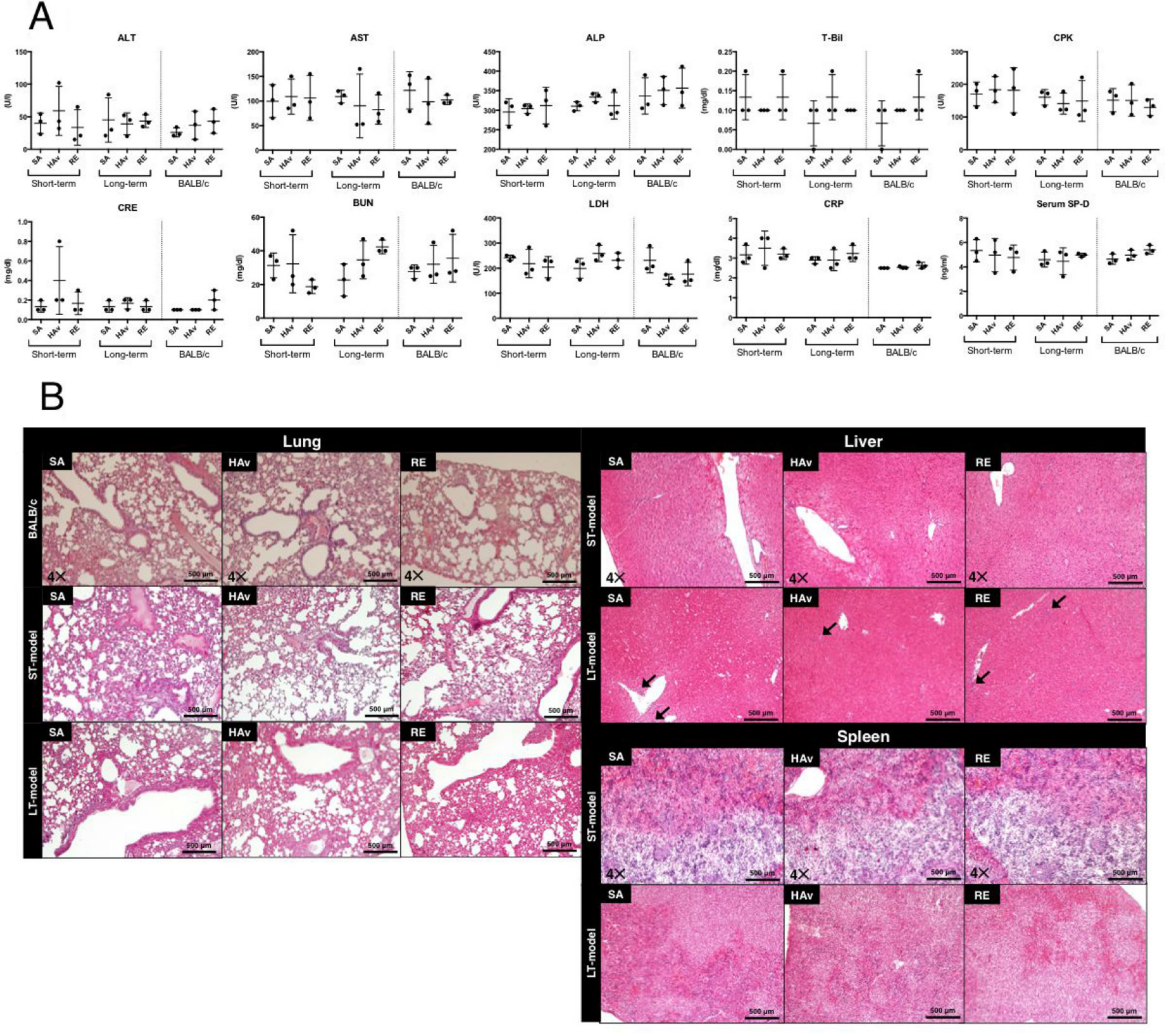
Blood biochemical data and lung, liver, and spleen histopathology of the short-term and long-term humanized mouse models and BALB/c mice Each animal group was inoculated with the toxicity reference vaccine (RE), hemagglutinin split vaccine (HAv), or saline (SA), and 16 h after the vaccination, serum was collected to obtain blood biochemical data on blood urea nitrogen (BUN), creatinine (CRE), creatinine kinase (CPK), alanine aminotransferase (ALT), aspartate aminotransferase (AST), total bilirubin (T-Bil), lactate dehydrogenase (LDH), alkaline phosphatase (ALP), C-reactive protein (CRP), and serum surfactant protein-D (SP-D) (**A**). The lungs, liver, and spleen were collected for the histopathological examination (**B**). Multiple 4-μm-thick slices were stained with hematoxylin and eosin (H&E). The arrows indicate infiltration by leukocytes.

**Figure 3:**
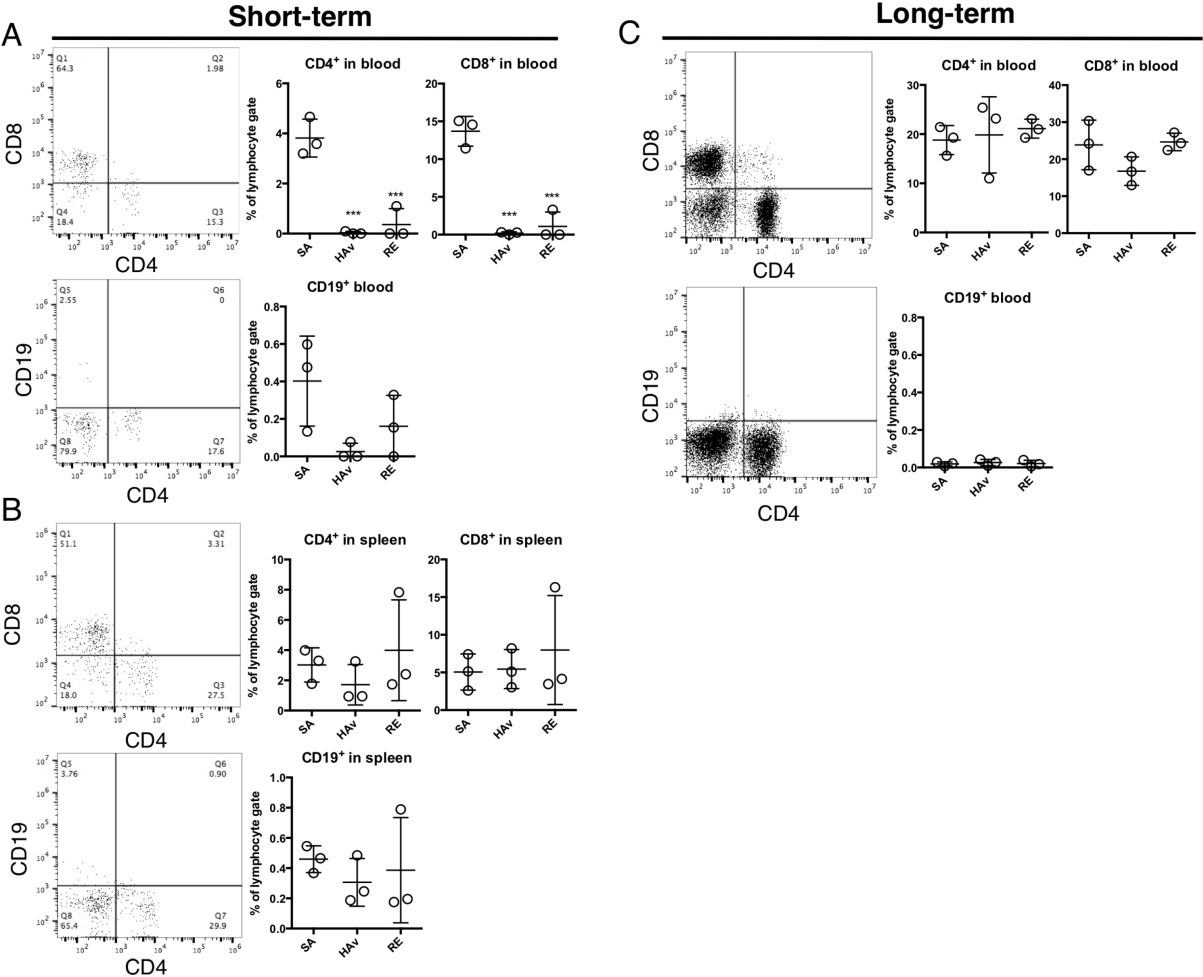
Retention of human immune cells in the lungs of humanized mouse models Representative dot plots of CD4^+^ T cells, CD8^+^ T cells, and B cells (CD19^+^) in blood (**A**) and the spleen (**B**) of the ST-model, and blood (**C**) of the LT-model. The humanized mice were vaccinated according to the scheme in Figure 1, and 16 h after the vaccination, lungs were collected and subjected to the flow-cytometric analyses. Dot plots indicate the rate of lymphocyte gating in each cell subset. Each circle represents a result from an individual animal, and horizontal bars denote mean ± SD. The difference from the saline (SA)-treated group was statistically significant at ^***^P < 0.001 in Dunnett's test.

**Figure 4:**
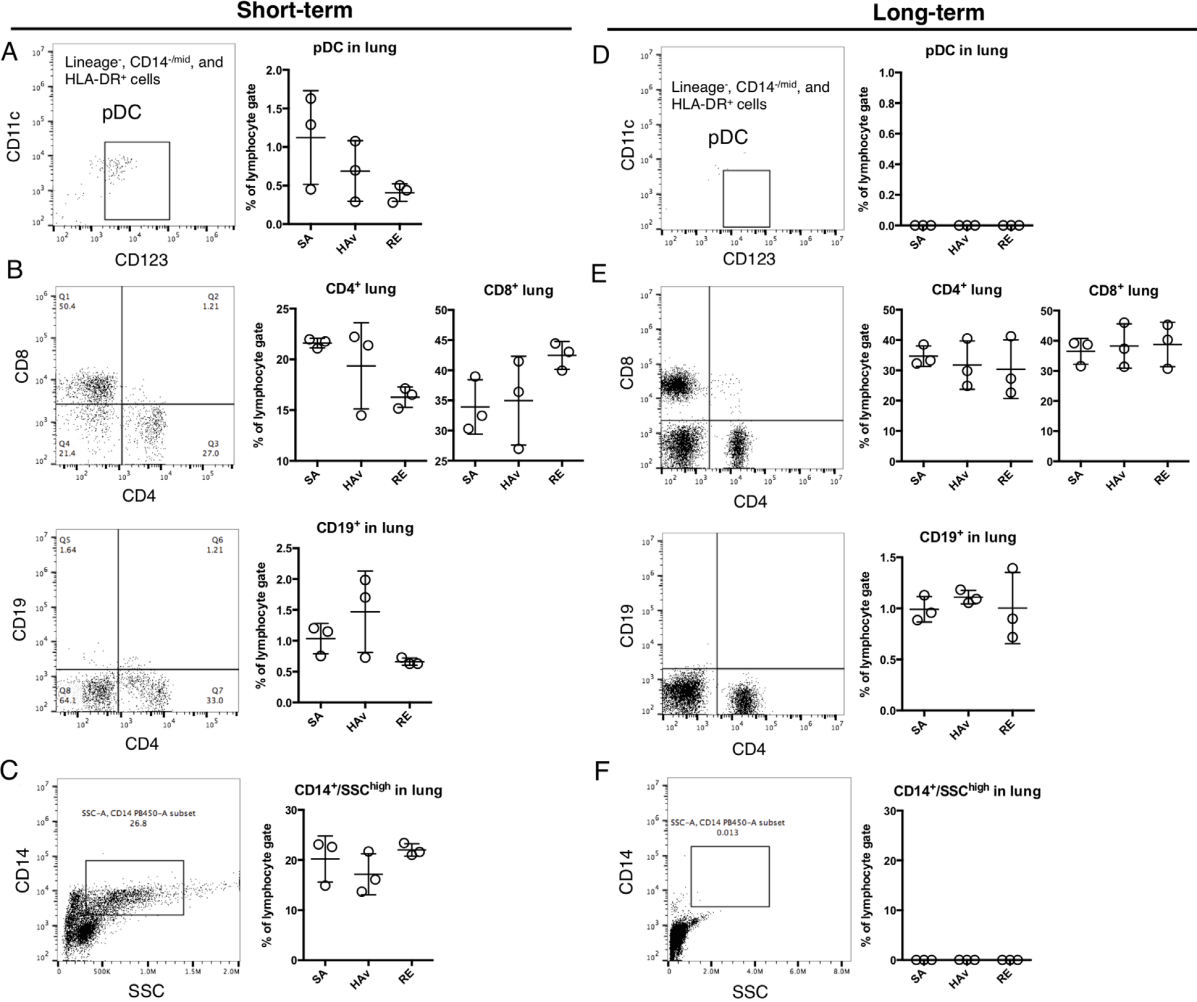
Retention of human immune cells in the blood and spleen of humanized mouse models Representative dot plots of pDCs (**A** and **D**) (lineage^−^, HLA-DR^+^, CD14^−/mid^, CD14^−^, and CD123^+^), (**B** and **E**) CD4^+^ T cells, CD8^+^ T cells, B cells (CD19^+^), and (**C** and **F**) monocytes (CD14^+^/SSC^high^) in the ST-model (first letters) and LT-model (second letters). The humanized mice were vaccinated according to the scheme in Figure 1, and 16 h after the vaccination, lungs were collected and applied to the flow-cytometric analyses. Dot plots indicate the rate of lymphocyte gating in each cell subset. Each circle represents a result from an individual animal, and horizontal bars indicate mean ± SD.

**Figure 5:**
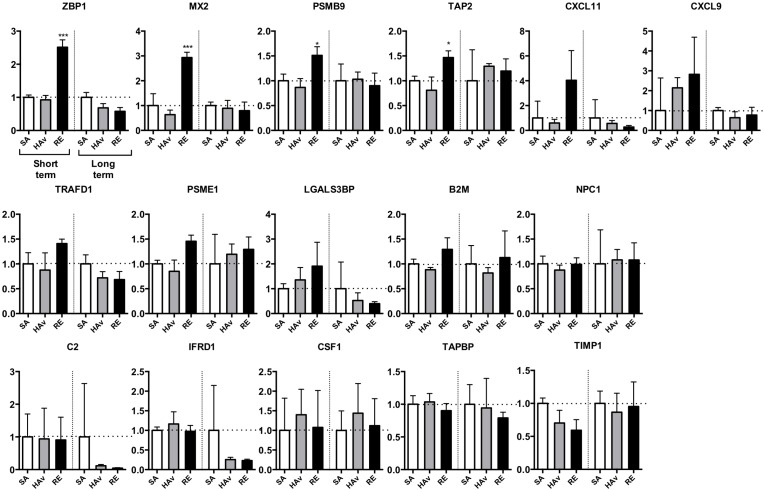
Expression analyses of human marker genes in lungs from vaccinated humanized mouse models The two humanized mouse models were inoculated with the toxicity reference vaccine (RE), hemagglutinin split vaccine (HAv), or saline (SA), and 16 h after the vaccination, lungs were collected and studied by the Quanti Gene Plex (QGP) assay, as described in the Methods. Data are mean ± SD (*n* = 3) and represent expression levels relative to *HPRT-1*. The difference from the saline (SA)-treated group was statistically significant at ^**^P < 0.01 and ^***^P < 0.001 in Dunnett's test.

**Figure 6:**
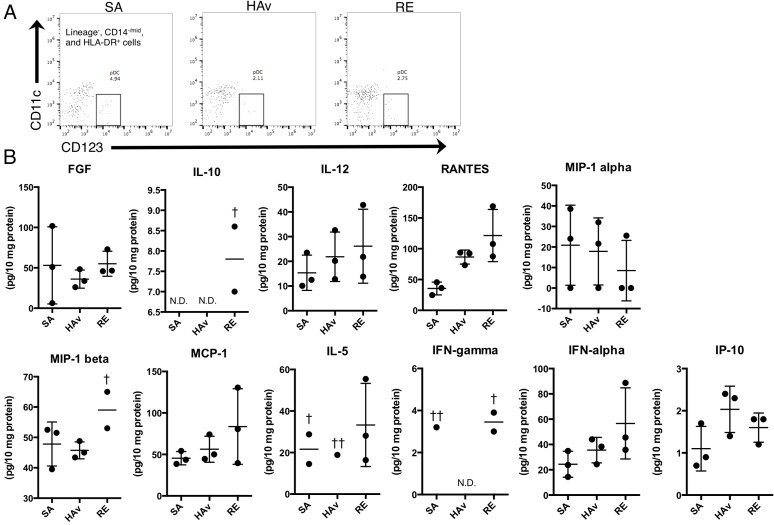
Human cytokine profiles of lung lysates from vaccine-inoculated humanized mice The ST-model mice were inoculated with the toxicity reference vaccine (RE), hemagglutinin split vaccine (HAv), or saline (SA), and 16 h after the vaccination, lungs were collected and subjected to lysate preparation and flow-cytometric analyses. (**A**) As shown in dot plots of pDCs in lungs from vaccinated mice; panel (**B**) presents the levels of human cytokines in lung lysates. The values of cytokine concentrations were adjusted for total protein contents. Each circle represents a result from an individual animal, and horizontal bars indicate mean ± SD. Symbol ^†^ indicates “not detected in 1/3 of the animals,” symbol ^††^ indicates “not detected in 2/3 of the animals,” and N.D. means “not detected in any animals in the group in question”.

To assess the lesions in organs, we analyzed blood biochemical and histopathological changes of the following organs: the spleen, liver, and lungs. Furthermore, to verify the differences in toxic reactions between the humanized mice and wild-type mice, BALB/c mice were also subjected to blood biochemical analyses and lung histopathological examination. Serum blood urea nitrogen (BUN) and creatinine (CRE) served as indicators of kidney functions; creatinine kinase (CPK) served as an indicator of muscle damage; alanine aminotransferase (ALT), aspartate aminotransferase (AST), alkaline phosphatase (ALP) and total bilirubin (T-Bil) were used as indicators of liver damage; lactate dehydrogenase (LDH) served as an indicator of tissue damage; C-reactive protein (CRP) was an indicator of inflammatory reactions; and surfactant protein-D (SP-D) served as an indicator of lung damage. The blood biochemical analyses showed that no significant changes were induced by vaccination in either the ST- or LT-model (Figure 2A). In addition, the same results were observed when BALB/c mice were studied as test animals (Figure 2A). In the liver, infiltrating cells, which are presumed to be predominantly lymphocytes but may include smaller numbers of neutrophils and/or macrophages, were observed in saline (SA)-treated, hemagglutinin split vaccine (HAv)-treated, or toxicity reference vaccine (RE)-treated LT-models (Figure 2B). Necrotic or apoptotic cell death was not observed in most cases. In other organs, no necrotic cell death, apoptotic cell death, inflammatory reactions, or other histopathological changes were induced by vaccination in either the ST- or LT-model (Figure 2B). In addition, the same results were observed in lungs when BALB/c mice were used as test animals (Figure 2B). As described in the Materials and Methods section, the donor of PBMCs used in the histopathological analyses (Figure 2B) was differed from the donor for other experiments (e.g., Figures 1, 2A, and 3–6). The engraftment of human PBMCs in ST- and LT-models used for the histopathological examination was confirmed by FACS analyses as shown in Supplementary Figure 2. Thus, noticeable lesions or inflammatory reactions in the liver, kidneys, lungs, and spleen were not caused by any vaccinations in the present safety evaluation method.

### FACS analyses of engrafted human cells in blood and the spleen

To test whether human PBMCs were retained and repopulated peripheral blood (PB) and spleen in the humanized mice, flow-cytometric analyses of the lungs at 16 h after vaccination were performed on both models. In the ST-model, CD4^+^ or CD8^+^ T cells were detected in PB; however, CD19^+^ cells were scarce (Figure 3A). Of note, numbers of CD4^+^ or CD8^+^ T cells were significantly decreased by the HAv or RE inoculation as compared with SA-treated mice (Figure 3A). Likewise, numbers of CD19^+^ cells decreased in response to an HAv or RE inoculation but not significantly (Figure 3A). In the spleen, CD4^+^ or CD8^+^ T cells were also detected, but CD19^+^ cells were rare (Figure 3B). The numbers of these cells were not altered by vaccinations (Figure 3B). In the LT-model in PB, CD4^+^ or CD8^+^ T cells were detected, but almost no CD19^+^ cells were detected (Figure 3C). In addition, repopulation levels of CD4^+^ or CD8^+^ T cells were higher than those in the ST-model (Figure 3C). In PB and the spleen, pDCs and CD14^+^ and SSC^high^ cells were not detected in either model (data not shown). These results indicated that both the ST- and LT-model retained human T cells systemically.

### FACS analyses of engrafted human cells in the lungs

To determine whether human PBMCs were retained and repopulated the lungs in the humanized mice, flow-cytometric analyses of the lungs at 16 h after vaccination were conducted in both models. In the ST-model, lineage^−^, CD14^−/mid^, HLA-DR^+^, CD123^+^, and CD11c^low^ cells indicating pDCs were detected (Figure 4A). pDCs numbers showed a tendency to decrease in RE-inoculated mice. CD4^+^ or CD8^+^ T cells, CD19^+^ cells (indicating B cells), and CD14^+^/ SSC^high^ cells were also detected; however, CD19^+^ cells were scarce. The retained levels of these lymphocytes were not changed by vaccination (Figure 4B and 4C). These results indicated that the ST-model retained human immune cells including not only T cells but also pDCs and monocytes in the lungs. In contrast, in the LT-model, pDCs and the CD14^+^/SSC^high^ population were not detected (Figure 4D and 4F). CD4^+^ or CD8^+^ T cells showed a much higher proportion than that in the ST-model (Figure 4B and 4E); however, CD19^+^ cells were scarce just as in the ST-model (Figure 4E). These results suggested that the LT-model manifested a high rate of retention of T cells but not of pDCs and monocytes.

### Analyses of expression of marker genes in lungs

To test whether human marker genes expressed in humanized mice are suitable for safety evaluation, the lungs were subjected to gene expression analyses. In the ST-model, the four marker genes, *ZBP1*, *Mx2*, *PSMB9*, and *TAP2*, were significantly upregulated in RE-inoculated mice as compared with SA-treated mice (Figure 5). The expression levels of *CXCL11*, *CXCL9*, *TRAFD1*, and *PSME1* also increased in response to the RE inoculation but not significantly (Figure 5). *LGALS3BP* was upregulated by both HAv and RE inoculations (Figure 5). The other genes were not upregulated in response to HAv or RE inoculations (Figure 5). These results indicated that *ZBP1*, *MX2*, *PSMB9*, *TAP2*, *CXCL11*, *CXCL9*, *TRAFD1*, and *PSME1* may be potential marker genes of human immunogenicity and immunotoxic reactions in the ST-model. By contrast, in the LT-model, no elevation of the marker gene expression levels was observed (Figure 5). These results meant that the LT-model is not suitable for safety evaluation of an influenza vaccine by means of the marker genes. The mouse marker genes were also analyzed in the ST-model (Supplementary Figure 3) and LT-model (Supplementary Figure 4). The mouse marker gene expression levels increased in response to RE inoculation, but this reaction was weaker in non-PBMC-engrafted NOG mice (Supplementary Figure 3).

### Human cytokine analyses

To test whether human PBMCs retained in humanized mice can produce cytokines and chemokines, human cytokine levels in a lung lysate from the ST-model were analyzed at 4 h after vaccination by a multiplex cytokine assay. At this time point, pDCs were still present in lungs (Figure 6A). In total, 11 of 30 cytokines and chemokines were detected (Figure 6B). Among these, regulated on activation normal T cell expressed and secreted (RANTES), macrophage inflammatory protein (MIP)-1b, and interferon (IFN)-a tended to increase in RE-inoculated mice as compared with SA-treated mice (Figure 6B). These results suggested that reconstituted human PBMCs in the lungs of the ST-model could produce cytokines and chemokines.

## DISCUSSION

In this study, the two humanized mouse models were devised, and their suitability for vaccine safety evaluation was assessed. The present study shows that the ST-model retained human pDCs and CD14^+^ SSC^high^ cells in lungs (Figure 4A and 4D). This phenomenon was not observed in the LT-model (Figure 4D and 4F). In addition, CD4^+^ or CD8^+^ T cells were systemically retained (Figure 3A and 3B) in the ST-model. The advantage of this model is easy preparation without any cell isolation or enrichment procedure for PBMCs. Furthermore, because there is no need for long-term breeding for PBMC reconstitution after the transplant, testing can be finished within a short period.

Whole-virion inactivated influenza vaccines, including RE, are known to cause leukopenic reactions in mice [19–21]. By contrast, both the ST- and LT-model did not show leukopenic reactions in PB. The retention rates of human pDCs in PB were extremely low in both the ST-model and LT-model (Figure 3A and 3C). pDCs can produce type I IFNs and are needed for the development of leukopenia induced by a whole-virion inactivated influenza vaccine in mice [21], suggesting that a lack of pDCs in PB may cause the absence of leukopenic toxicity in the present model. Accordingly, it is necessary to further improve the humanized mouse model for leukopenic toxicity assessment.

Although leukopenia was undetectable, increased expression levels of the marker genes in response to RE inoculation were detectable in the ST-model (Figure 5). According to these results, it is likely that the sensitivity of the marker genes is higher than that of the leukopenic reaction for detection of RE-mediated immunogenicity in the ST-model. The LT-model, however, which completely lacks pDCs and CD14^+^/SSC^high^ cells in lungs (Figure 4D), did not reveal any elevation of the marker gene expression levels in lungs (Figure 5). These results suggest that pulmonary pDCs and CD14^+^/SSC^high^ cells may be important for the upregulation of marker genes.

The marker genes include type I IFN-related genes, as typified by *ZBP1* [22], *MX2* [23], *CXCL11* [24], and *CXCL9* [25] (Figure 5). As discussed above, type I IFNs also play a role in the leukopenic toxicity induced by a whole-virion inactivated influenza vaccine [21]. These effects may be deeply involved in pDC-mediated reactions. pDCs can respond to a whole-virion inactivated influenza vaccine via Toll-like receptor (TLR) 7 and then rapidly secrete IFN-*γ*. In the present study, pDCs were retained in the lungs of the ST-model (Figure 5), and IFN-a in lung lysates showed a tendency for an increase in response to RE inoculation (Figure 6B), suggesting that the ST-model reflects human pDC-mediated reactions. Besides, the expression levels of mouse marker genes increased in response to RE inoculation (Supplementary Figure 3). In addition, these responses were attenuated in non-PBMC-engrafted NOG mice (Supplementary Figure 3), suggesting that mouse marker genes might have responded via human PBMC- and cytokine-mediated reactions.

The marker gene sets include type I IFN-related or -inducible genes as typified by *IRF7*, *LGALS3BP*, and *LGALS9* [26–28]. In addition, *MX2*, *TAP2*, and *PSMB9* induction by a type I IFN has been reported [29, 30]. Influenza virus infection, which can induce type I IFNs, is known to contribute to leukopenic toxicity, implying that these genes may partially participate in influenza vaccine–induced leukopenic toxicity [21]. Furthermore, excessive type I IFN signaling results in uncontrolled inflammation, and TNF-related apoptosis-inducing ligand–death receptor 5-mediated epithelial cell death has been reported [30]. Overall, type I IFN–related genes may partially reflect the influenza infection–related leukopenic toxicity and inflammatory reactions.

Additionally, IFN-γ-inducible *PSME1* is a component of immunoproteasomes, which play a role in the generation of MHC-I-presented peptides [31]. This reaction is important for activation of cytotoxic-T-lymphocyte–mediated suppression of infectious spread of influenza viruses [31]. These studies led us to wonder whether marker genes may be able to evaluate not only toxic effects but also reactogenicity and effectiveness.

Although the marker genes can be informative because of their known functions, animal studies (rats and mice) have been limited in terms of applicability to humans judging by the obtained results. In the presented models, both human PBMC-mediated reactions and informative data on the expression levels of marker genes are seen. Thus, the proposed human-PBL model may offer the benefit of safety evaluation of influenza vaccines in humans. In addition, the possible benefit of using the human-PBL model is that it can provide information about both human and mouse marker genes. In particular, the lungs in the human-PBL model contain human PBMCs, mouse PBMCs, and mouse parenchymal cells (such as epithelial cells). As shown in Supplementary Figures 3 and 4, the mouse genes were responsible for vaccination effects. Furthermore, these elevated expression levels of mouse marker genes were not observed in non-PBMC-engrafted NOG mice, suggesting that PBMC-mediated reactions can increase expression levels of the mouse marker genes (Supplementary Figures 3 and 4). The responsible marker genes in humans were found to be limited to 8 out of 16, suggesting that analysis of only PBMCs (i.e., a PBMC culture system) means that only about a half or less of all the marker genes are affected, resulting in less information. Thus, analyzing the mouse marker gene expression levels may provide more robust information.

In the human-PBL model, as compared to one study on rats [3], significant increases in the expression of only 4 genes were observed among human genes (Figure 5). In the previous studies on rats, we observed changes in gene expression levels across whole lung tissues, which included lung parenchymal cells, lymphocytes, and DCs; however, expression levels of human genes in humanized mice are detected only among the genes expressed in PBMCs. Thus, human genes in humanized mice cannot reflect the gene expression patterns of parenchymal cells in the lungs. In fact, analyses of mouse genes in the lungs of humanized mice revealed upregulation of many marker genes as in the case of rat studies (Supplementary Figure 3) in contrast to the expression levels of human genes (Figure 5). Furthermore, elevated mouse gene expression levels were lower in non-PBMC-engrafted NOG mice than in PBMC-engrafted NOG mice. From these data, we concluded that the expression of lung marker genes increased not only in lymphocytes and DCs but also in lung parenchymal cells.

In conclusion, in this study, we for the first time developed a short-term model involving pDCs and CD 14^+^/SSC^high^ cells in the lungs, and this humanized mouse model may be useful for the human safety evaluation of influenza vaccines by means of human marker genes. In addition, our present data indicate that some of the marker genes can be applicable to humans, and we expect that this information can form the basis for the development of novel methods for assessment and prediction of human safety of influenza vaccines. Because commercially available PBMCs were used in this study, we could not show whether a vaccination history or infection history can be useful for this evaluation system. In the future, if it becomes possible to evaluate the history of a PBMC donor (i.e., the presence or absence of adverse effects of vaccination or a history of vaccinations and infections), such data will make this model more robust.

## MATERIALS AND METHODS

### Animals and an ethics statement

Female 6- to 7-week-old NOD/Shi-*scid-IL2r*g*^null^* (NOG; formal name, NOD.*Cg-prkdcscidil2rgtm1Sug/*Jic) mice were purchased from *In-Vivo* Science, Inc. (Kawasaki, Japan), and female 6- to 7-week-old BALB/c mice (16–20 g) were obtained from SLC (Tokyo, Japan), and maintained under specific pathogen-free conditions. All the mice were housed in rooms maintained at 23 ± 1°C and 50% ± 10% relative humidity, in a 12 h light/dark cycle, and were provided with sterilized food and water *ad libitum*. The mice were subjected to experiments at the age of 7 weeks and were acclimated for at least 7 days before the experiments. All the animal experiments were performed according to the Declaration of Helsinki and the guidelines of the Institutional Animal Care and Use Committee of the National Institute of Infectious Diseases (NIID), Tokyo, Japan. The study protocol was approved by the Institutional Animal Care and Use Committee of NIID.

### Engraftment of PBMCs

Human PBMCs from one healthy donor (24 years old, male adult Caucasian) were obtained from AllCells (Alameda, CA, USA) and were subjected to the experiment shown in Figures 1 and 2 (except for panel B) and Figures 3–6. Human PBMCs from one healthy donor (47 years old, female adult Caucasian) were acquired from Lonza (Basel Switzerland) and were used for histopathological assays in the ST-model as presented in Figure 2B. Human PBMCs from one healthy donor (27 years old, male adult Caucasian) were obtained from AllCells (Alameda, CA, USA), and were used in the histopathological experiment in the LT-model as shown in Figure 2B.

Prior to the engraftment, the PBMCs were resuspended in the RPMI 1640 medium supplemented with 10% of fetal calf serum (FCS) at a density of 4 × 10^7^ cells/ml and transplanted via the tail vein into NOG mice at 4 × 10^7^ cells/mouse to create the ST-model. To set up the LT-model, 10^7^ PBMCs were intraperitoneally injected into NOG mice. The mice were subjected to the vaccination experiment at 3 weeks after the PBMC engraftment. This is because more than 3 weeks later, the mice will develop graft-versus-host disease according to our preliminary study (data not shown). The experiments on human blood samples were approved by the Institutional Ethics Committee on Human Experimentation and were conducted in accordance with the Ethical Guidelines for Medical and Health Research Involving Human Subjects in Japan.

### Flow cytometry

The red blood cells were lysed with an ammonium chloride solution, and mononuclear cells were prepared as single-cell suspensions in 5% FCS-supplemented PBS. In particular, lungs and spleen were treated with 1 mg/ml type IV collagenase (Thermo Fisher Scientific, Waltham, MA) in Hanks' balanced salt solution at 37°C for 30 min. The digestion reactions were stopped by adding EDTA (10 mM) and placement on ice. Mononuclear cells were incubated for 30 min at 4°C in the dark with the appropriate antibodies (Supplementary Table 1). After washing with 5% FCS–supplemented PBS, the stained cells were studied on a CytoFLEX Flow Cytometer (Beckman Coulter Inc., CA, USA). The acquired data were analyzed in the FlowJo software (TreeStar, San Carlos, CA. The gating strategies are illustrated in Supplementary Figure 1.

### Vaccines and vaccinations

RE is a national toxicity reference issued by NIID (Japan). RE is a lyophilized whole-virion preparation of an inactivated influenza virus and consists of the three strains of inactivated whole-virions: A/Newcaledonia/20/99 (H1N1), A/Hiroshima/52/2005 (H3N2), and B/Malaysia/2506/2004. RE is employed as the toxicity reference in the leukopenic toxicity test (LTT) in Japan [2]. The influenza A virus (A/New Caledonia/20/99; H1N1) HAv was kindly provided by Dr. Hideki Asanuma (National Institute of Infectious Diseases, Tokyo, Japan). Like RE, HAv was reconstituted with SA to prepare solutions of 15 μg hemagglutinin (HA) per 0.5 ml. The amount of the HA antigen was chosen according to the LTT method in JMR [2]. Each solution was intraperitoneally injected into the mice (0.5 ml/mouse) according to the LTT method and the test of mouse body weight decrease in JMR [2]. At 16 h after the vaccination, their blood was collected via the inferior vena cava. At the same time, the lungs and spleen were immediately collected. The number of WBCs was determined with an automatic hemocytometer, Celltac MEK-6450 (Nihon Kohden, Tokyo, Japan). For the cytokine analyses, the collection of lungs and blood was performed at 4 h after vaccinations.

### Blood biochemical analyses

The serum BUN, CRE, CPK, ALT, AST, T-Bil, LDH, ALP, and CRP levels were measured by means of a DRI-CHEM (Fujifilm, Tokyo, Japan). The SP-D levels were analyzed with the Quantikine Human/Mouse SP-D ELISA Kit (Minneapolis, MN). One study indicates that serum SP-D levels increase in response to acute lung inflammation in mice [32]; therefore, we analyzed serum SP-D levels as a lung injury biomarker.

### Histopathological examination

The lungs, liver, and spleen were excised and fixed in 10% neutral-buffered formalin. The fixed samples were dehydrated in graded alcohol washes and embedded in paraffin. Serial sections of 4 μm thickness were stained with hematoxylin and eosin (H&E) and examined under a microscope for routine histopathological examination. Images were acquired by means of an Olympus BX53 microscope (Tokyo, Japan).

### Preparation of lung lysates and expression analyses of marker genes

The lung lysates were prepared, and QuantiGene Plex (QGP) was performed to analyze the expression levels of marker genes in lungs as described in our previous studies [5–6]. Detection probes for human (Table 1) and mouse marker genes (Supplementary Table 2) have already been confirmed to not cross-react with each other in a preliminary study on RNA from human PBMCs and lung tissue from mice (data not shown). The expression levels of the marker genes and hypoxanthine-guanine phosphoribosyl transferase (*HPRT-1)* were quantified simultaneously, and the ratio of each marker gene expression to that of *HPRT-1* was calculated.

**Table 1:** Human marker genes and internal standard gene for the safety evaluation of influenza vaccines

Symbol	Official Full Name	Accession
*CXCL11*	C-X-C motif chemokine ligand 11	NM_005409.4
*CXCL9*	C-X-C motif chemokine ligand 9	NM_002416.2
*ZBP1*	Z-DNA binding protein 1	NM_030776.2
*MX2*	MX dynamin like GTPase 2	NM_002463.1
*IRF7*	Interferon regulatory factor 7	NM_004031.2
*NPC1*	NPC intracellular cholesterol transporter 1	NM_000271.4
*TAPBP*	TAP binding protein	NM_003190.4
*CSF1*	colony stimulating factor 1	NM_000757.5
*TIMP1*	TIMP metallopeptidase inhibitor 1	NM_003254.2
*TRAFD1*	TRAF-type zinc finger domain containing 1	NM_001143906.1
*LGALS3BP*	galectin 3 binding protein	NM_005567.3
*PSMB9*	proteasome subunit beta 9	NM_002800.4
*C2*	complement C2	NM_000063.5
*TAP2*	transporter 2, ATP binding cassette subfamily B member	NM_000544.3
*IFRD1*	interferon related developmental regulator 1	NM_001550.3
*PSME1*	proteasome activator subunit 1	NM_006263.3
*B2M*	beta-2-microglobulin	NM_004048.2
*HPRT1*	hypoxanthine phosphoribosyltransferase 1	NM_000194.2

*HPRT1* served as an internal standard gene.

### Human cytokine analyses

For preparation of a lung lysate, a lung was homogenized with Ca^2+^- and Mg^2+^-free PBS, and then 0.05% Triton X-100 was added to a portion of the homogenized lung lysate, and the sample was incubated for 2 h at 4°C with intermittent mixing. At the end of incubation, the supernatant was collected by centrifugation. Cytokine analyses were performed using the Cytokine 30-Plex Human Panel Kit (Thermo Fisher Scientific, Waltham, MA).

### Statistical analyses

For multiple comparisons, one-way analysis of variance followed by Dunnett's multiple-comparison test were performed. The statistical analyses were conducted in GraphPad Prism 6 (GraphPad Software, La Jolla, CA). Data with *P* < 0.05 were considered statistically significant.

## SUPPLEMENTARY MATERIALS FIGURES AND TABLES



## Abbreviations

ALP: Alkaline phosphatase
ALT: alanine aminotransferase
AST: aspartate aminotransferase
BUN: blood urea nitrogen
CPK: creatinine kinase
CRE: creatinine
CRP: C-reactive protein
DCs: dendritic cells
HAv: hemagglutinin split vaccine
LDH: lactate dehydrogenase
JMR: Minimum Requirements for Biological Products Guidelines of Japan
LT-model: long-term model
PBLs: peripheral blood lymphocytes
pDCs: plasmacytoid DCs
PBMCs: peripheral blood mononuclear cells
SA: saline
RE: toxicity reference vaccine
ST-model: short-term model
T-Bil: total bilirubin
WBC: white blood cell
SP-D: serum surfactant protein-D
