# Supplementary material for: Development of a preclinical humanized mouse model to evaluate acute toxicity of an influenza vaccine
Source: Oncotarget. 2018 May 25;9(40):25751–63. doi: 10.18632/oncotarget.25399 (PMC5995229; doi:10.18632/oncotarget.25399)
Supplement: Supplementary file 1 [file oncotarget-09-25751-s001.pdf]

Development of a preclinical humanized mouse model to evaluate acute toxicity of an influenza vaccine

SUPPLEMENTARY MATERIALS

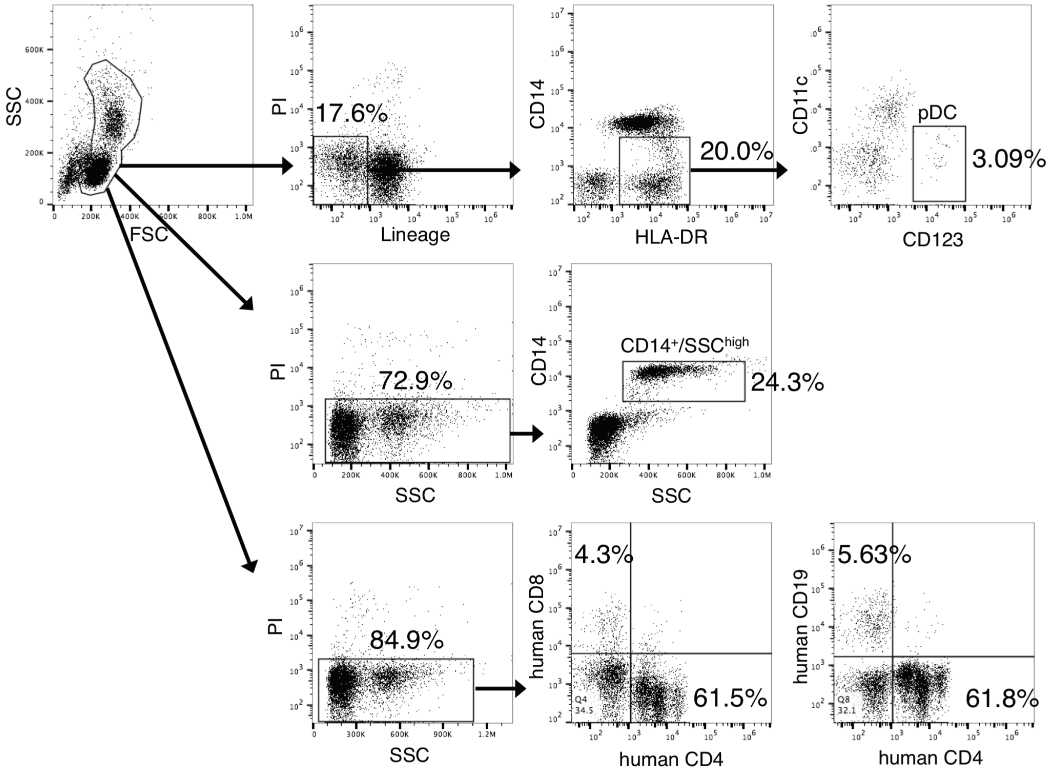

Supplementary Figure 1: Representative gating strategies and characterizing lymphocyte, monocyte and dendritic cell frequencies in peripheral blood mononuclear cells (PBMC). Representative dot plots of (top) plasmacytoid dendritic cells (Lineage$^-$, HLA-DR$^+$, human CD14$^{-/mid}$, human CD11c$^-$, and human CD123$^+$), (Middle) monocytes (human CD14$^+$/SSC^{high}) and (bottom) human CD4$^+$ T cells, human CD8$^+$T cells, B cells (human CD19$^+$) in PBMC.

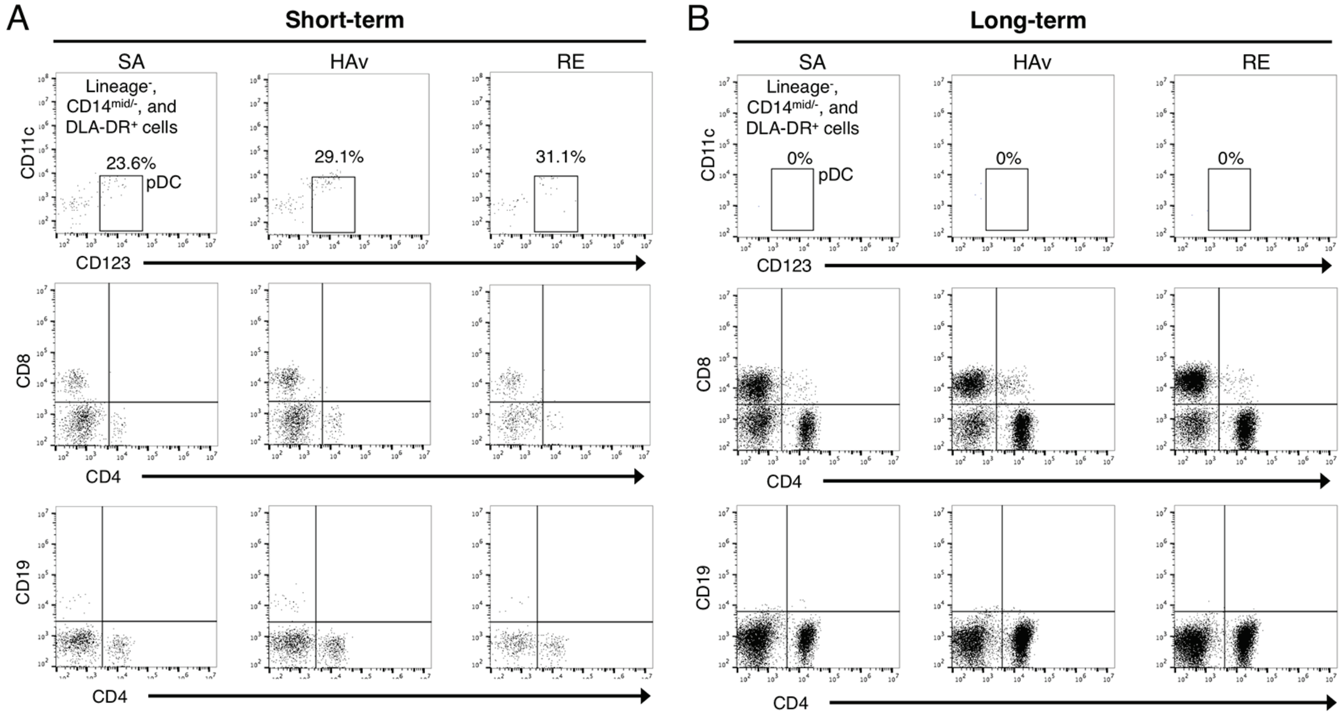

Supplementary Figure 2: Retaining of human immune cells in lung of humanized mouse models used for histopathological analyses. Representative dot plots of (top) plasmacytoid dendritic cells (Lineage$^-$, HLA-DR$^+$, human CD14$^{-/mid}$, human CD11c$^-$, and human CD123$^+$), (Middle) human CD4$^+$ T cells, human CD8$^+$T cells, and (bottom) human CD4$^+$ T cells and B cells (human CD19$^+$) in PBMC. The left panel indicates the result from the short-term model (**A**) and right panel indicates the result from the long-term model (**B**).

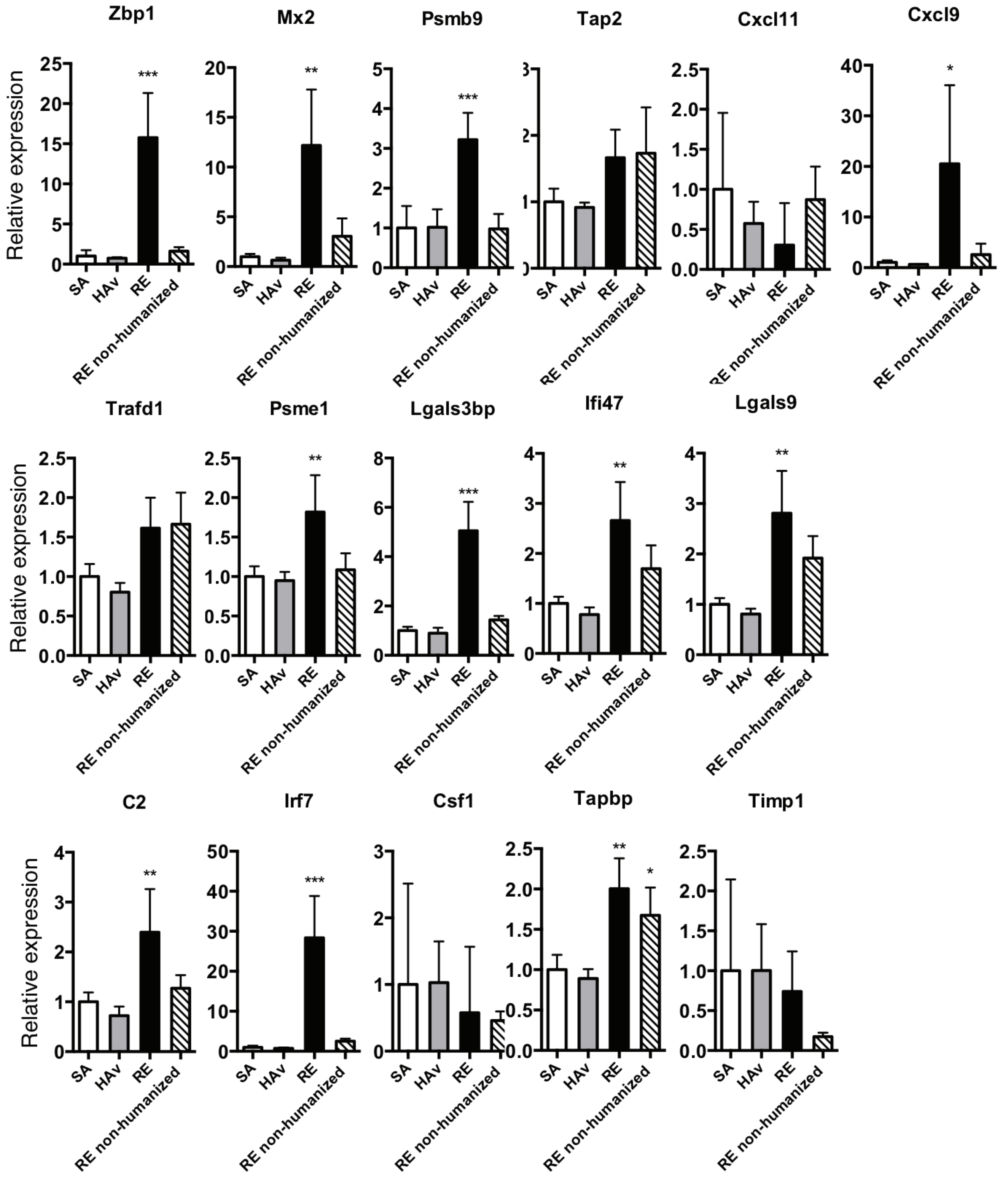

Supplementary Figure 3: Mouse marker genes expression analyses in lung from vaccinated short-term humanized mouse models. The short-term humanized mouse models were inoculated with toxicity references vaccine (RE), hemagglutinin split vaccine (HAv) or saline (SA), and 16hrs after the vaccination, lungs were collected and used for Quanti Gene Plex (QGP) assay, as described in the Methods. Data are the mean ± SD ($N = 3$). Data represent expression levels relative to beta-actin. The difference between SA-treated group were statistically significant at $^{*}P < 0.01$, $^{**}P < 0.01$ and $^{***}P < 0.001$ by Dumnett's test.

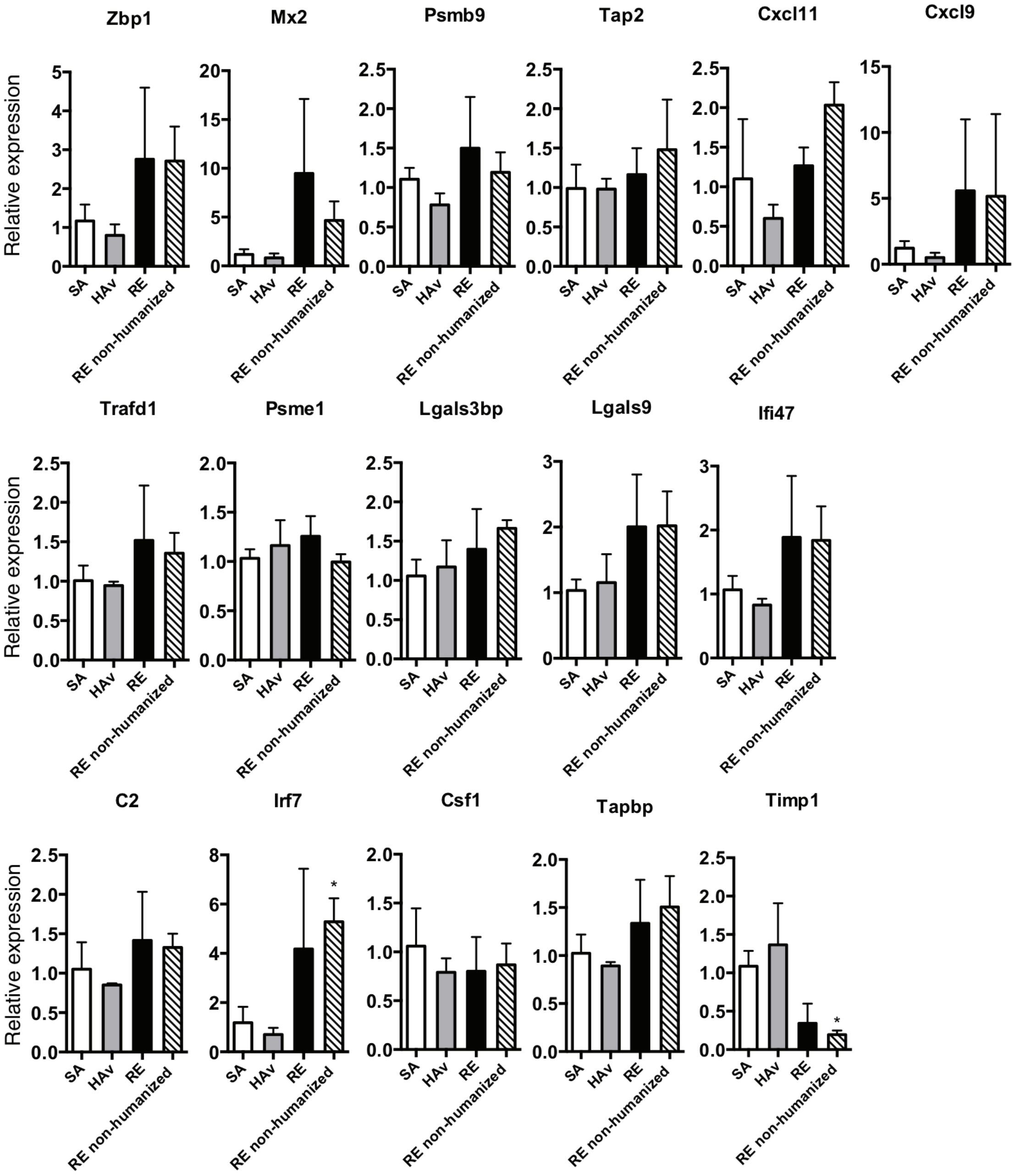

Supplementary Figure 4: Mouse marker genes expression analyses in lung from vaccinated long-term humanized mouse models. The long-term humanized mouse models were inoculated with toxicity references vaccine (RE), hemagglutinin split vaccine (HAv) or saline (SA), and 16 hrs after the vaccination, lungs were collected and used for Quanti Gene Plex (QGP) assay, as described in the Methods. Data are the mean ± SD ($N = 3$). Data represent expression levels relative to beta-actin. The difference between SA-treated group were statistically significant at $^{*}P < 0.01$ by Dumnett's test.

Supplementary Table 1: The list of antibodies used for flow cytometry

Antibody	Source	Clone	Conjugate
Human CD3	eBioscience	HIT3a	phycoerythrin
Human CD20	eBioscience	2H7	phycoerythrin
Human CD56	eBioscience	CMSSB	phycoerythrin
Human HLA-DR	eBioscience	LN3	fluorescein isothiocyanate
Human CD19	eBioscience	HIB19	phycoerythrin
Human CD8a	eBioscience	OKT8	allophycocyanin
Human CD14	eBioscience	61D3	allophycocyanin
Human CD4	eBioscience	OKT4	fluorescein isothiocyanate
Human CD14 for pDC	BioLegend	61D3	pacific blue
Human CD11c	eBioscience	3.9	allophycocyanin-eFluor780
Human CD123	BD Pharmingen	7G3	allophycocyanin

Supplementary Table 2: Mouse marker genes for the safety evaluation of influenza vaccines

Symbol	Official full name	Accession number
Cxcl11	Chemokine (C-X-C motif) ligand 11	NM_019494
Cxcl9	Chemokine (C-X-C motif) ligand 9	NM_008599
Zbp1	Z-DNA binding protein 1	NM_021394
Mx2	MX dynamin-like GTPase 2	NM_013606
Irf7	Interferon regulatory factor 7	NM_016850
Lgals9	Lectin, galactoside-binding, soluble, 9	NM_010708
Ifi47	Interferon gamma inducible protein 47	NM_008330
Tapbp	TAP binding protein (tapasin)	NM_001025313
Csf1	Colony stimulating factor (macrophage)	NM_007778
Timp1	Tissue inhibitor of metalloproteinase 1	NM_001044384
Trafd1	TRAF type zinc finger domain containing 1	NM_001163470
Lgals3bp	Lectin, galactoside-binding, soluble, 3 binding protein	NM_011150
Psmb9	Proteasome (prosome, macropain) subunit, beta type, 9	NM_013585
C2	Complement component 2	NM_013484
Tap2	Transporter 2, ATP-binding cassette, sub-family B (MDR/TAP)	XM_006525355
Ifrd1	Interferon-related developmental regulator 1	NM_013562
Psme1	Proteasome (prosome, macropain) activator subunit 1	NM_011189
Ngfr	Nerve growth factor receptor	NM_033217
